# Chronic Deep Brain Stimulation of the Hypothalamic Nucleus in Wistar Rats Alters Circulatory Levels of Corticosterone and Proinflammatory Cytokines

**DOI:** 10.1155/2013/698634

**Published:** 2013-10-23

**Authors:** Juan Manuel Calleja-Castillo, Dora Luz De La Cruz-Aguilera, Joaquín Manjarrez, Marco Antonio Velasco-Velázquez, Gabriel Morales-Espinoza, Julia Moreno-Aguilar, Maria Eugenia Hernández, Lucinda Aguirre-Cruz, Lenin Pavón

**Affiliations:** ^1^Laboratory of Neuroimmunoendocrinology, National Institute of Neurology and Neurosurgery “Manuel Velasco Suárez”, Avenida Insurgentes Sur 3877, La Fama, Tlalpan, 14269 Mexico City, DF, Mexico; ^2^Laboratory of Reticular Formation Physiology, National Institute of Neurology and Neurosurgery “Manuel Velasco Suárez”, Avenida Insurgentes Sur 3877, La Fama, Tlalpan, 14269 Mexico City, DF, Mexico; ^3^Department of Pharmacology, School of Medicine, National Autonomous University of Mexico, P.O. Box 70-297, Coyoacan, 04510 Mexico City, DF, Mexico; ^4^Department of Psychoimmunology, National Institute of Psychiatry “Ramón de la Fuente”, Calzada México-Xochimilco 101, Col. San Lorenzo Huipulco, Tlalpan, 14370 Mexico City, DF, Mexico

## Abstract

Deep brain stimulation (DBS) is a therapeutic option for several diseases, but its effects on HPA axis activity and systemic inflammation are unknown. This study aimed to detect circulatory variations of corticosterone and cytokines levels in Wistar rats, after 21 days of DBS-at the ventrolateral part of the ventromedial hypothalamic nucleus (VMHvl), unilateral cervical vagotomy (UCVgX), or UCVgX plus DBS. We included the respective control (C) and sham (S) groups (*n* = 6 rats per group). DBS treated rats had higher levels of TNF-*α* (120%; P < 0.01) and IFN-*γ* (305%; P < 0.001) but lower corticosterone concentration (48%; P < 0.001) than C and S. UCVgX animals showed increased corticosterone levels (154%; P < 0.001) versus C and S. UCVgX plus DBS increased IL-1*β* (402%; P < 0.001), IL-6 (160%; P < 0.001), and corsticosterone (178%; P < 0.001 versus 48%; P < 0.001) compared with the C and S groups. Chronic DBS at VMHvl induced a systemic inflammatory response accompanied by a decrease of HPA axis function. UCVgX rats experienced HPA axis hyperactivity as result of vagus nerve injury; however, DBS was unable to block the HPA axis hyperactivity induced by unilateral cervical vagotomy. Further studies are necessary to explore these findings and their clinical implication.

## 1. Introduction

The clinical use of deep brain stimulation (DBS) has increased in recent years [1]. This treatment has become a therapeutic option for pathologies that are associated with chronic pain and movement disorders [2] as well as for refractory depression [3] or epilepsy [4]. Such patients can be treated with direct electrical stimulation at the vagus nerve [5, 6] or at deep nuclei of the hypothalamus [4, 7–9]. The use of DBS in humans entails the implantation of a generator of electric current (commonly under the collarbone) and bilateral electrodes that transmit a continuous current to precise stereotaxic coordinates into the brain [10]. 

Although DBS was initially considered to mimic a lesion, the mechanism by which this therapy exerts its effects *in vivo* is complex and incompletely understood [11]. The electric stimulation of nerves triggers depolarization of the membrane in the associated neurons [12]. Accordingly, DBS devices induce axonal activation and neuronal inhibition in animal models [2, 13, 14]. Theoretically, these effects evoke activity in areas that received axonal projections that are adjacent to the stimulating electrode [15, 16]. The reported changes on neurotransmitters levels at anatomical area in which DBS is applied [17, 18] support this concept. 

The hypothalamic nuclei are regions of interest to assess the interaction that exists between the nervous system and the immunological response since these hypothalamic nuclei anatomically connect two primary neural routes that modulate the inflammatory response: the HPA axis [19] and the sympathetic nervous system [20]. Additionally, both routes regulate the peripheral concentrations of the chief stress hormones cortisol, adrenaline, and noradrenaline [21].

The vagus nerve participates in a neural circuit that modulates innate immunity. This circuit is activated by cytokines and other inflammatory mediators in tissues that trigger afferent action potentials that travel by the vagus nerve. The ascending information is relayed to brainstem nuclei that control efferent neural signals that are transmitted back to the periphery in the form of action potentials via the vagus nerve [22]. This information is sent to the spleen and other cytokine-producing organs, where cytokine expression is inhibited by a molecular mechanism that requires the *α*7 subunit of the acetylcholine nicotinic receptor. The negative feedback by the motor arc of the inflammatory reflex prevents the damage of excessive innate immune responses—this circuit is known as the cholinergic anti-inflammatory pathway [22].

Until recently, no clinical or experimental study had described changes in vagus nerve function after application of DBS in hypothalamic nuclei. In epileptic patients, vagus nerve stimulation (VNS) reduces systemic levels of IL-6 and TNF-*α* but increases those of IL-10 and TGF-*β* [5, 23]. Such changes might be linked to its therapeutic effectiveness. Conversely, VNS elicits an anti-inflammatory response in several animal models of chronic and acute inflammatory syndromes [24–26]. VNS also regulates serum cortisol concentrations in patients [5] and corticosterone in rodents [27]. Vagal afferents represent a functional link between peripheral cytokine release and activation of the HPA axis. For example, subdiaphragmatic vagotomy blocks adrenocorticotropic hormone (ACTH) and corticosterone production when low doses of cytokines are administered intraperitoneally or intravenously [28–30]. However, activation of the HPA axis with higher doses of cytokines might involve additional neural and humoral pathways [28, 31, 32].

Activation of nerve fibers (i.e., once a nerve action potential is elicited) by chemicals or electrical stimulation establishes nerve-to-nerve or nerve-to-brain tissue communication. The solitary tract nucleus (STN)—the main terminal of vagal nerve afferents in the CNS—makes anatomic connections with corticotrophin-releasing cells in the paraventricular nucleus of the hypothalamus [33, 34]. Imaging studies have detected activation of the hypothalamus on electrical stimulation of the vagal nerve [35, 36]. Accordingly, Hosoi et al. reported elevation of serum corticosterone and ACTH on electrical stimulation of the vagal nerve in anesthetized rats [37]. These findings support a model in which electrical stimulation of the vagal nerve under experimental conditions activates brain structures that constitute the HPA axis. However, changes in the HPA axis or vagus nerve function due to DBS of hypothalamic nuclei have not been reported. 

Our group hypothesized that electrical stimulation of hypothalamic nuclei during DBS would have immunoendocrine effects. Thus, our aim was to assess the immunological and endocrinological effects of chronic DBS (21 days) of the ventrolateral section of the ventromedial hypothalamic nucleus (VMHvl) in Wistar rats with or without unilateral cervical vagotomy (UCVgX). We measured the serum levels of corticosterone, IL-1*β*, TNF-*α*, IL-6, and IFN-*γ* in the absence of epileptogenic or antigenic stimuli.

## 2. Methods

### 2.1. Animals

Male Wistar rats, weighing 250–300 g at the time of surgery, were used. Animals were housed in individual cages at 25°C on a 12 h light/dark illumination cycle (light from 8 AM to 8 PM) and had free access to food and water. All animal procedures were performed as per the following guidelines: (i) the Neurology and Neurosurgery National Institute's Ethical Code for the care and use of laboratory animals, (ii) Mexican guidelines for the production, care, and use of laboratory animals (NOM-062-ZOO-1999), and (iii) the National Institutes of Health Guide for the Care and Use of Laboratory Animals (NIH Publications number 80-23, revised in 1978). All efforts were made to minimize animal suffering and reduce the number of animals that was used.

Animals were randomly assigned to one of the following experimental groups, each containing 6 rats: control without treatment (C), sham vagal surgery (S), unilateral cervical vagotomy (UCVgX), UCVgX plus electrodes without electric stimulation (UCVgX + E/WES), sham plus electrodes without electric stimulation (S + E/WES), DBS at VMHvl (DBS), and unilateral cervical vagotomy plus DBS (UCVgX + DBS). All animals were allowed to acclimate to the experimental conditions for 5 days before treatment.

### 2.2. Unilateral Cervical Vagotomy

Vagotomy was performed under general anesthesia with ketamine (80 mg/kg ip) and xylazine (5 mg/kg im) as described [38]. Briefly, a midline incision of 1.5 cm was made in the anterior neck to localize the right vagus trunk. The right vagus nerve was separated carefully from the carotid artery and cut, and the skin was sutured with mononylon. Sham (S) rats were operated on to expose the vagal trunk, but the vagus nerve was not cut. We performed unilateral cervical vagotomy to analyze the effects of partial blockage of vagal function.

### 2.3. DBS

Monopolar stainless steel electrodes of 100 *μ*m, entirely insulated except for 0.25 mm at the tip (World Precision Instruments Inc., USA), were implanted bilaterally in anesthetized animals using a stereotaxic frame (Stoelting Co., USA) to reach the VMHvl (coordinates: 3.2 mm anterior, 0.6 mm lateral, and 9.6 mm ventral to the bregma) [39]. A 100 *μ*m stainless steel electrode was placed on the skull bone as a reference. All electrodes were welded to a female connector. Anchor screws were set in the skull, and the assembly was secured with dental cement. 

After surgery, the animals were allowed to recover in their home cage with food and water *ad libitum* and treated with buprenorphine (0.1 mg/kg ip) every 8 h over 3 days to minimize pain. After 1 week, animals were placed in acrylic boxes (30 × 30 × 30 cm) with a Plexiglas Arena and connected to a Grass S88 stimulator (Model PSIU6; Grass Quincy Mass, USA) by a flexible insulated cable that permitted free movement. Rats were allowed to explore the area for 30 min (habituation) before electrical stimulation with a fixed frequency of 50 Hz with an average current intensity of 550 *μ*A for 30 s. The threshold of electrical stimulation was screened by increasing the current gradually until a change in behavior (sleep, excessive scratching, explorative behavior, sniffing, and ipsilateral ptosis) was observed, at which point current was fixed. VMHvl electrodes current intensity was adjusted for each animal to induce the change of behavior. The electrode which gave reliable behavioral response at a lower current intensity was chosen as the stimulating electrode for further experiments [40]. 

DBS treatment was administered during 30 minutes in which 30 cycles of alternate 30 s of electrical stimulation and 30 s of rest along 21 days were applied. Unstimulated control animals (WES) were connected to the stimulator without current being applied. 

### 2.4. Quantification of IL-1*β*, TNF-*α*, IL-6, and IFN-*γ* by ELISA

After 21 days of treatment, blood samples (2.5 mL) were taken by cardiac puncture from anesthetized rats. Serum was separated and stored at −70°C until analysis. Rat cytokines were quantified using the ELISA Development System Kit and monoclonal antibodies against TNF-*α* (Cat. RTA00), IFN-*γ* (Cat. RIF00), IL-1*β* (Cat. RLB00), or IL-6 (Cat. R6000B) (all from R&D Systems, USA). 

Antibodies were used to precoat a microplate before incubation with the samples (diluted 1 : 4) in triplicate. After wash steps, the appropriate enzyme-linked antibodies were added to the wells. Optical densities were quantified at 492 nm after addition of substrate and stop solutions. All incubations were performed at room temperature. Ranges of detection (pg/mL) were determined using standards as follows: IL-1*β* = 31.2–1000, TNF-*α* = 12.5–800, IL-6 = 62.5–2000, and IFN-*γ* = 31.2–1000. Intra- and interassay variability were less than 5% and 8%, respectively.

### 2.5. Quantification of Corticosterone by Radioimmunoassay

Total corticosterone from serum samples was quantified according to Keppler and Decker [41] in triplicate using the commercially available Coat-A-Count Rat Corticosterone radioimmunoassay (RIA) kit (Siemens) as per the manufacturer's instructions.

### 2.6. Statistical Analysis

Homogeneity of variance test for each molecule was followed by one-way ANOVA. Bonferroni's test was used as a *post hoc* test to compare responses between groups. Statistical analyses were performed using GraphPad Prism, version 6.00 for Mac OS X (GraphPad Software, USA). The statistical significance was established at *P* < 0.05.

## 3. Results

**Figure 1:**
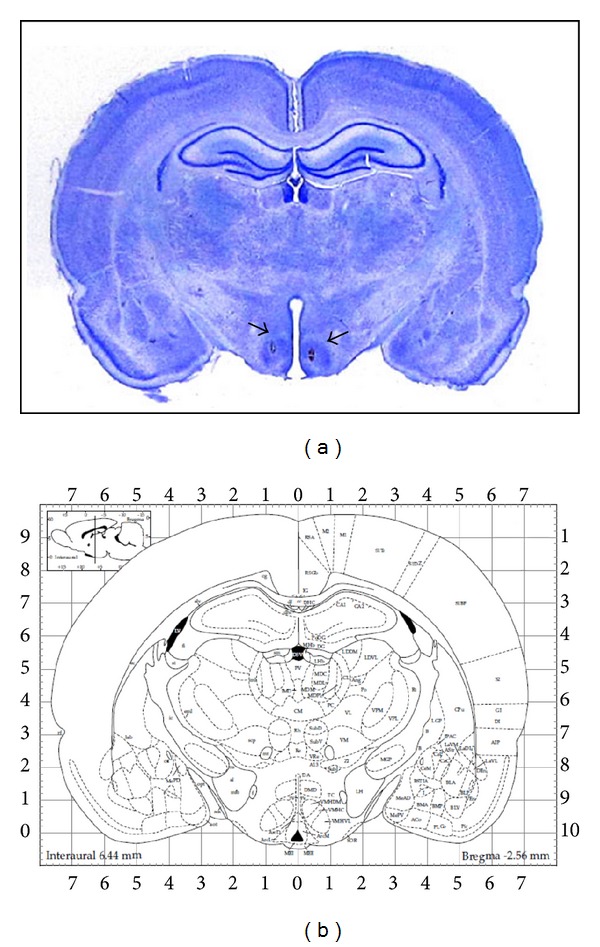
Sites of electrode implantation for cerebral electrical stimulation in rats. (a) Photograph of electrodes implanted in the ventrolateral portion of hypothalamic ventromedial nuclei, VMHvl (bregma −2.6 mm)in a coronal section of brain rat, stained with the Nissl Technique (5x). (b) Schematic representation of the VMHvl [39].

At the end of the experiments, localization of the electrodes in the VMHvl was confirmed with Nissl Technique-stained coronal sections (Figure 1). The circulatory concentrations of cytokines were quantified in serum from DBS-stimulated rats throughout the 21 days of treatment. All data are reported in pg/mL. 

### 3.1. IL-1*β*

**Figure 2:**
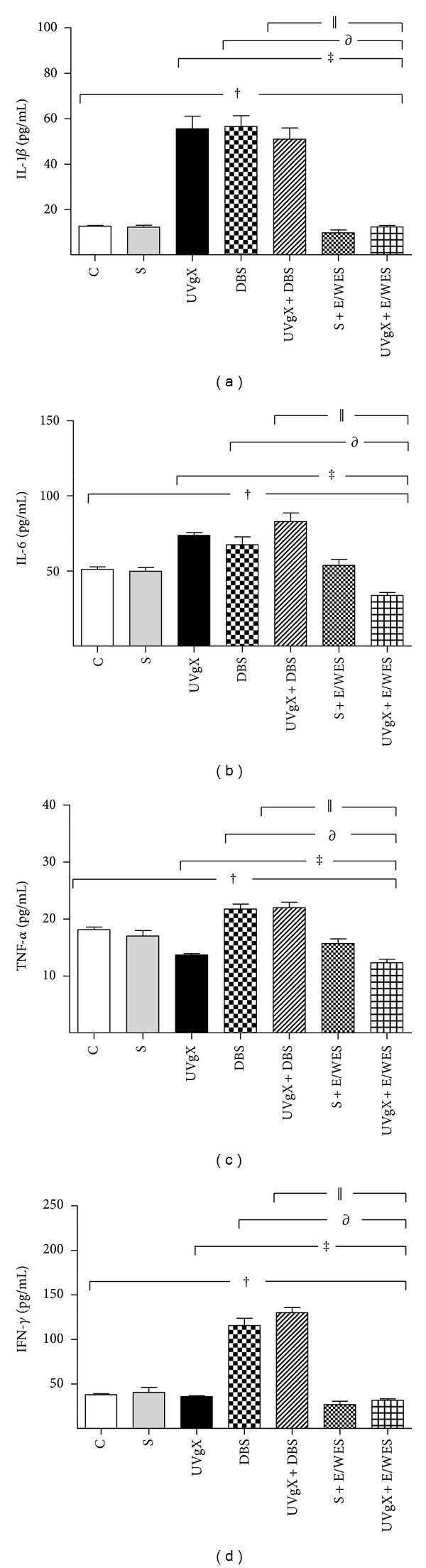
Effect of unilateral vagotomy and deep brain stimulation of hypothalamic nucleus on cytokine levels in Wistar rats. Serum IL-1*β*, IL-6, TNF-*α* and IFN-*γ*, levels were detected by capture ELISA assay. Animals received the following experimental procedures; Sham (S): animals were operated to expose the vagal trunk, without cutting the vagus nerve. Sham plus electrodes/without electric stimulation (S + E/WES). Unilateral vagotomy (UVgX): right vagus nerve was carefully separated from the carotid artery and cut. Unilateral vagotomy plus electrodes/without electric stimulation (UVgX + E/WES). Deep brain stimulation (DBS): animals were implanted bilaterally with two electrodes. Unilateral vagotomy plus deep brain stimulation (UV + DBS). Animals without treatment (C). Experimental groups (*n* = 6 rats) were followedup 21 days and showed significant differences compared to C, S, UVgX, DBS, and UVgX + DBS. Data are expressed as mean ± SE. The differences between means were evaluated with one-way ANOVA with Bonferroni's post hoc. Statistical significance was attributed when *P* < 0.05. ^†^ *P* < 0.001 C or S versus UVgX, DBS, UVgX + DBS, S + E/WES, and UVgX + E/WES groups; ^‡^ *P* < 0.001 UVgX versus DBS, UVgX + DBS, S + E/WES, and UVgX + E/WES groups; ^∂^ *P* < 0.001 DBS versus UVgX  +  DBS, S  +  E/WES, and UVgX + E/WES groups;^||^ *P* < 0.001 UVgX + DBS, S + E/WES, and UVgX + E/WES groups versus C.

IL-1*β* serum concentrations differed significantly between groups (*F* = 82.21, df = 1,6; *P* < 0.0001). Unilateral vagotomy (UCVgX), DBS of the VMHvl (DBS), and their combination (UCVgX + DBS) increased IL-1*β* concentrations compared with the control (C) and sham (S) groups (55.5 ± 12, 56.5 ± 11, and 50.9 ± 11 versus 12.65 ± 1 and 12.21 ± 2, resp.; *P* < 0.001). There were no significant differences between UCVgX, DBS, and UCVgX + DBS animals. Levels in the sham, sham plus electrodes without electric stimulation (S + E/WES), and UCVgX plus electrodes without electric stimulation (UCVgX + E/WES) groups did not differ from those in the C group (Figure 2(a)).

### 3.2. TNF-*α*

TNF-*α* levels differed between treatments (*F* = 82.21, df = 1,6; *P* < 0.0001). The UCVgX and UCVgX + E/WES groups had lower levels compared with the C and S groups (13.7 ± 1 and 12.3 ± 1 versus 18 ± 2 and 17.02 ± 2; *P* < 0.001). Conversely, DBS increased TNF-*α* concentrations versus C rats (21.8 ± 2 versus 18 ± 2; *P* < 0.001). UCVgX + DBS rats had significantly higher TNF-*α* levels than UCVgX animals (22 ± 2 versus 13.7 ± 0.5; *P* < 0.01). Levels in S and S + E/WES rats did not differ compared with C rats (Figure 2(b)).

### 3.3. IL-6

IL-6 differed significantly between groups (*F* = 23.11, df = 6,1; *P* < 0.0001). IL-6 increased after UCVgX, DBS, and UCVgX + DBS treatments (79.8 ± 15, 83 ± 13, and 67.4 ± 12 versus 51 ± 6 in control rats; *P* < 0.001). UCVgX + E/WES reduced IL-6 levels compared with UCVgX (31.7 ± 3 versus 51 ± 6; *P* < 0.01). IL-6 after S and S + E/WES treatments was similar to levels in C rats (Figure 2(c)).

### 3.4. IFN-*γ*

IFN-*γ* levels differed significantly between treatments (*F* = 120.8, df = 6,1; *P* < 0.0001). DBS and UCVgX + DBS increased circulatory IFN-*γ* levels compared with C and S (115.5 ± 18 and 129.8 ± 13 versus 37.8 ± 6 or 40.5 ± 12; *P* < 0.001). Similarly, IFN-*γ* rose after UCVgX + DBS versus UCVgX (129.8 ± 13 versus 35.7 ± 2; *P* < 0.001). However, concentrations in UCVgX-treated rats were unchanged in S and C, indicating that DBS upregulates IFN-*γ* despite damage to the vagus nerve. Sham treatment had no effect versus C (Figure 2(d)).

### 3.5. Corticosterone

**Figure 3:**
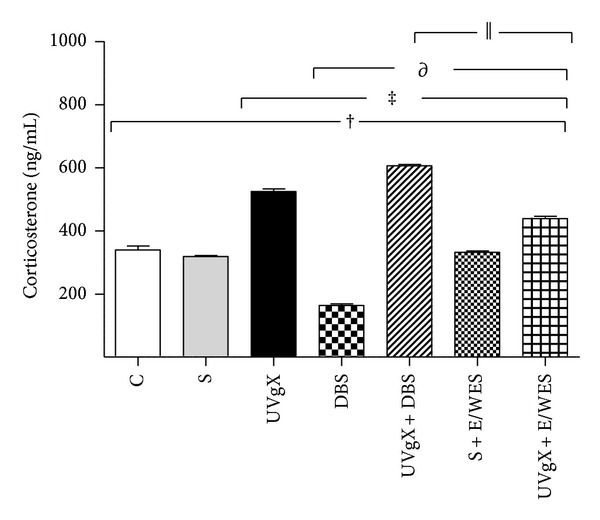
Effect of unilateral vagotomy and deep brain stimulation of hypothalamic nucleus on corticosterone levels in Wistar rats. Corticosterone determination was by RIA assay. Wistar rats received the following experimental procedures; Sham (S): animals were operated on to expose the vagal trunk, without cutting the vagus nerve. Sham plus electrodes/without electric stimulation (S + E/WES). Unilateral vagotomy (UVgX): right vagus nerve was carefully separated from the carotid artery and cut. Unilateral vagotomy plus electrodes/without electric stimulation (UVgX + E/WES). Deep brain stimulation (DBS): animals were implanted bilaterally with two electrodes. Unilateral vagotomy plus deep brain stimulation (UV + DBS). Animals without treatment (C). Experimental groups (*n* = 6 rats) were followedup 21 days and showed significant differences compared to C, S, UVgX, DBS and, UVgX + DBS. Data are expressed as mean ± SE. The differences between means were evaluated with one-way ANOVA with Bonferroni's post hoc. Statistical significance was attributed when when *P* < 0.05. ^†^ *P* < 0.001 C or S versus UVgX, DBS, UVgX + DBS, S + E/WES, and UVgX + E/WES; ^‡^ *P* < 0.001 UVgX versus, DBS, UVgX + DBS, S + E/WES, and UVgX + E/WES; ^∂^ *P* < 0.001 DBS versus UVgX + DBS, S + E/WES, and UVgX + E/WES; ^||^ *P* < 0.001 UVgX + DBS, S + E/WES, and UVgX + E/WES versus C.

Corticosterone levels differed between groups (*F* = 28.97, df = 6,1; *P* < 0.005). DBS-treated rats had lower concentrations than C animals (164.1 ± 11 versus 339.3 ± 31; *P* < 0.001). In contrast, UCVgX- and UCVgX + DBS-treated animals had higher levels than C or S rats (524.8 ± 2 and 606.6 ± 10 versus 339.3 ± 31 and 318.9 ± 8; *P* < 0.001). Groups C, S, and S + E/WES did not differ (Figure 3).

## 4. Discussion

The immune response cells constitutively express receptors for hormones, neurotransmitters, and cytokines [42], being susceptible to changes in the concentration of these soluble mediators. Our results show that the application of DBS or UCVgX leads to changes in circulatory levels of corticosterone and proinflammatory cytokines. 

### 4.1. DBS Effects

A major issue influencing corticosterone and circulatory cytokine profiles reported in both treatments is the neuroendocrine, immune network. The HPA axis function is upregulated by proinflammatory cytokines through the brain receptors for these soluble molecules, expressed mostly at hypothalamus [43]. This stimulation induces a rise in circulatory levels of glucocorticoids that decreases the inflammatory systemic effects induced by cytokines and diminishes the release of CRH at hypothalamus, generating a negative feedback loop. In this study DBS and UVgX treatments induce an increase in circulatory levels of cytokines but only DBS treatment presents a significant decrease in corticosterone levels which are associated with functionality of HPA axis.

Serum glucocorticoid concentration is an accepted indicator of HPA axis activation [44]. As of the preparation of this paper, two studies in patients with Parkinson disease (PD) have described the effects of DBS at the subthalamic nucleus on the HPA axis. First, Novakova et al. reported significantly decreased cortisol levels from months 2 to 12 compared with baseline (*P* < 0.01, corrected) [45]. In the second report, Seifried et al. reported that 24 h mean cortisol levels decreased 6 months after electrode implantation surgery in PD patients (pre-OP 9.06 ± 2.63 versus post-OP 7.025 ± 3.46; *P* = 0.05) [46]. Similarly, de Koning et al. reported that obsessive-compulsive disorder patients that received DBS at nucleus accumbens showed a decrease of median urinary excretion of free cortisol [47]. Our results showing that Wistar rats that received chronic DBS at VMHvl had lower serum corticosterone levels are consistent with those reports. 

The authors of those reports agree that DBS modulates HPA axis directly or indirectly through neural connections between the anatomical areas stimulated by DBS and the hypothalamus [45–47]. Moreover, Ballanger et al. reported that the effects of DBS are not restricted to a single anatomic location, since subthalamic nucleus DBS drives subthalamic nucleus output in not only the immediate target region but also the remote and widespread areas of the basal ganglia, brainstem, cerebellum, and cortex [48].

Our study shows that chronic DBS at VMHvl, which is a part of hypothalamus, limits activation of the HPA axis, reducing the levels of corticosterone. The HPA axis inhibition by DBS may be generated by neuronal blockage. Electrical stimulation depolarizes the membrane of neurons, inducing action potentials and triggering neurotransmitters release from vesicles. Under normal conditions, neurons have a period of rest that allows membranes to repolarize. However, chronic electric simulation affects the depletion of neurotransmitters, consequently impeding neuronal activation [49].

On the other hand, this is the first report to analyze the effects of DBS on systemic inflammatory responses. Our results demonstrate that chronic DBS at VMHvl increases serum levels of the proinflammatory cytokines IL-1*β*, TNF-*α*, IL-6, and IFN-*γ*. Unfortunately, the design of our study did not allow us to elucidate the mechanisms by which DBS induces systemic changes in proinflammatory mediators. We speculate that the significant decrease of corticosterone levels in rats that received chronically DBS promotes the establishment of proinflammatory profile of cytokines in circulation. Variations in glucocorticoids like corticosterone can modulate lymphocyte proliferation and cytokine gene transcription [50]. High levels of glucocorticoids compromise the function of immune response and promote an anti-inflammatory response; on the contrary low levels of glucocorticoids promote the release of proinflammatory cytokines [50]. 

### 4.2. UCVgX Effects

Unilateral cervical vagotomy (UCVgX + DBS and UCVgX) groups increased the circulatory levels of corticosterone compared with the rest of the groups, which is consistent with previous reports showing that cervical vagotomy in pigeons upregulated serum corticosterone [51] and that subdiaphragmatic vagotomy in rats intensified the carbachol (cholinergic muscarinic and nicotinic agonist) effects over ACTH and corticosterone secretion [52]. Apparently the effects of vagotomy on corticosterone levels may result from disruption of the motor fibers (parasympathetic control of target organs and perhaps immune cells) and/or the disruption of the sensory fibers (over 70% of the vagus is sensory) that carry information from periphery to the central nervous system including immune system derived signals. Disruption of sympathovagal balance in response to real or perceived challenges/stressors leads to alteration in homeostasis and activation of the HPA axis [53]. Our results show that unilateral cervical vagotomy did not reproduce the proinflammatory cytokine pattern that is observed in DBS group. This may be caused by the significant increase of corticosterone levels in rats with UCVgX. 

### 4.3. UCVgX Plus DBS Effects

Both the HPA axis and the sympathetic nervous system regulate peripheral concentration of the main stress hormone cortisol [21]. Although the single effects of DBS and UCVgX produce opposite effects on serum cortisol concentration, the combined application of these treatments produces increases in corticosterone, IL-1*β*, and IL-6 levels. These results indicate that despite the existence of vagal afferents projects from brain steam to the solitary tract nucleus and hypothalamic nuclei, such as the VMHvl [33] DBS is unable to block the HPA axis hyperactivity induced by unilateral cervical vagotomy. This suggests that DBS neuronal blockage is not enough to reduce the anti-inflammatory response caused by UCVgX, because of either anatomic limitations on stimuli transmission or the existence of local compensation mechanisms. 

UVgX and UVgX plus E/WES groups showed similar circulatory levels of corticosterone, TNF-*α*, and IFN-*γ*. Interestingly, the levels detected for IL-1*β* and IL-6 were reduced in UVgX plus E/WES in comparison to UVgX. These results seem to be paradoxical, albeit they suggest the possibility that other compensatory mechanism could be involved in these phenomena. In healthy individuals the levels of proinflammatory cytokines are controlled by several mechanisms, including the activation of the IL-6 receptor and gp130 protein. When IL-6 binds to its receptor, a mechanism is triggered by blocking Janus kinase signal and activator of transcription (JAK/STAT) mediated transcription of IL-1*β* [54], decreasing its level in circulation [55]. Additionally, the manipulation of the vagus nerve is associated with modification on food intake, body weight gain, HPA axis activation, and glucose metabolism [56], these metabolic changes may modify the circulatory levels of IL-1*β* and IL-6 [57].

### 4.4. Limitations

There were certain limitations in this study. First, the effects in DBS were evaluated in a small sample (each group with *n* = 6) of single rat strain. Second, circulatory levels of acetylcholine, adrenaline, and noradrenaline should have been measured to determine the contribution of the vagus nerve during DBS. Despite that in the present work there are not direct experimental line of evidence of the immunological or endocrinological effects of DBS, the following two facts should be considered: first, there is a general consensus about the communication between central nervous and immune systems which regulates several physiological processes [43]. In this study we did not explore the specific source of cytokines because they could come from many different sources. Leukocytes, neural cells, fibroblasts, adipocytes, and endothelial cells can all release cytokines and many of them constitutively express receptors to hormones and neurotransmitters [42]. Hormones and neurotransmitters might modulate the profile and circulatory levels of cytokines in this way. The second fact is that the general phenomenon described in this paper has been previously reported in other systems. The release of soluble mediators by HPA axis or vagus nerve activation, such as cortisol and acetylcholine, respectively, has direct effects on circulatory levels and profile of cytokines in animal models and patients, similar to those seen in this work. Lastly, we did not perform a functional evaluation of the immune response, which would have required us to challenge the rats with infectious stimuli or cytokine administration during DBS or UVgX. Recent studies have reported that both the nature and the intensity of antigenic stimulation might affect the capability of hypothalamic nucleus to modulate HPA axis function, compared with the responses obtained during the stress stimulus [58]. All these issues will be considered in future studies.

## 5. Conclusions

Chronic DBS of the VMHvl impairs the HPA axis function, as reflected in the increase in circulatory levels of proinflammatory cytokines (IL-1*β*, TNF-*α*, IL-6, and IFN-*γ*) and decrease in corticosterone. UCVgX-treated rats experienced a HPA axis hyperactivity as a result of injury to the vagus nerve. DBS in UCVgX animals was unable to block the HPA axis hyperactivity induced by unilateral cervical vagotomy. These preliminary results suggest that immunity will be altered in patients who are treated with DBS, facilitating the development of strategies to prevent the secondary effects of DBS. Further studies are necessary to explore the clinical implications of these findings.

## References

[1] Benabid AL (2007). What the future holds for deep brain stimulation. *Expert Review of Medical Devices*.

[2] Benabid AL, Benazzous A, Pollak P (2002). Mechanisms of deep brain stimulation. *Movement Disorders*.

[3] Lozano AM, Giacobbe P, Hamani C (2012). A multicenter pilot study of subcallosal cingulate area deep brain stimulation for treatment-resistant depression: clinical article. *Journal of Neurosurgery*.

[4] Boon P, Raedt R, de Herdt V, Wyckhuys T, Vonck K (2009). Electrical Stimulation for the Treatment of Epilepsy. *Neurotherapeutics*.

[5] Majoie HJM, Rijkers K, Berfelo MW (2011). Vagus nerve stimulation in refractory epilepsy: effects on pro-and anti-inflammatory cytokines in peripheral blood. *NeuroImmunoModulation*.

[6] de Herdt V, Bogaert S, Bracke KR (2009). Effects of vagus nerve stimulation on pro- and anti-inflammatory cytokine induction in patients with refractory epilepsy. *Journal of Neuroimmunology*.

[7] Nishida N, Huang Z-L, Mikuni N, Miura Y, Urade Y, Hashimoto N (2007). Deep brain stimulation of the posterior hypothalamus activates the histaminergic system to exert antiepileptic effect in rat pentylenetetrazol model. *Experimental Neurology*.

[8] Marras CE, Rizzi M, Villani F (2011). Deep brain stimulation for the treatment of drug-refractory epilepsy in a patient with a hypothalamic hamartoma: case report. *Neurosurgical Focus*.

[9] Rahman M, Abd-El-Barr MM, Vedam-Mai V (2010). Disrupting abnormal electrical activity with deep brain stimulation: is epilepsy the next frontier?. *Neurosurgical Focus*.

[10] Rizvi SJ, Donovan M, Giacobbe P, Placenza F, Rotzinger S, Kennedy SH (2011). Neurostimulation therapies for treatment resistant depression: a focus on vagus nerve stimulation and deep brain stimulation. *International Review of Psychiatry*.

[11] Lozano AM, Dostrovsky J, Chen R, Ashby P (2002). Deep brain stimulation for Parkinson’s disease: disrupting the disruption. *The Lancet Neurology*.

[12] Rowbottom M, Susskind C (1984). *Electricity and Medicine: History of Their Interaction*.

[13] Dostrovsky JO, Lozano AM (2002). Mechanisms of deep brain stimulation. *Movement Disorders*.

[14] Vitek JL (2002). Mechnisms of deep brain stimulation: excitation or inhibition. *Movement Disorders*.

[15] McCracken CB, Grace AA (2009). Nucleus accumbens deep brain stimulation produces region-specific alterations in local field potential oscillations and evoked responses In vivo. *Journal of Neuroscience*.

[16] Sjöberg RL, Blomstedt P (2011). The psychological neuroscience of depression: implications for understanding effects of deep brain stimulation. *Scandinavian Journal of Psychology*.

[17] Sesia T, Bulthuis V, Tan S (2010). Deep brain stimulation of the nucleus accumbens shell increases impulsive behavior and tissue levels of dopamine and serotonin. *Experimental Neurology*.

[18] van Dijk A, Klompmakers AA, Feenstra MG, Denys D (2012). Deep brain stimulation of the accumbens increases dopamine, serotonin, and noradrenaline in the prefrontal cortex. *Journal of Neurochemistry*.

[19] Pavlov VA, Wang H, Czura CJ, Friedman SG, Tracey KJ (2003). The cholinergic anti-inflammatory pathway: a missing link in neuroimmunomodulation. *Molecular Medicine*.

[20] Tracey KJ (2002). The inflammatory reflex. *Nature*.

[21] de Kloet ER, Joëls M, Holsboer F (2005). Stress and the brain: from adaptation to disease. *Nature Reviews Neuroscience*.

[22] Andersson U, Tracey KJ (2012). Reflex principles of immunological homeostasis. *Annual Review of Immunology*.

[23] Corcoran C, Connor TJ, O’Keane V, Garland MR (2005). The effects of vagus nerve stimulation on pro- and anti-inflammatory cytokines in humans: a preliminary report. *NeuroImmunoModulation*.

[24] Wu R, Dong W, Cui X (2007). Ghrelin down-regulates proinflammatory cytokines in sepsis through activation of the vagus nerve. *Annals of Surgery*.

[25] Niederbichler AD, Papst S, Claassen L (2009). Burn-induced organ dysfunction: vagus nerve stimulation attenuates organ and serum cytokine levels. *Burns*.

[26] de Jonge WJ, van der Zanden EP, The FO (2005). Stimulation of the vagus nerve attenuates macrophage activation by activating the Jak2-STAT3 signaling pathway. *Nature Immunology*.

[27] de Herdt V, Puimege L, De Waele J (2009). Increased rat serum corticosterone suggests immunomodulation by stimulation of the vagal nerve. *Journal of Neuroimmunology*.

[28] Gaykema RPA, Dijkstra I, Tilders FJH (1995). Subdiaphragmatic vagotomy suppresses endotoxin-induced activation of hypothalamic corticotropin-releasing hormone neurons and ACTH secretion. *Endocrinology*.

[29] Fleshner M, Goehler LE, Hermann J, Relton JK, Maier SF, Watkins LR (1995). Interleukin-1*β* induced corticosterone elevation and hypothalamic NE depletion is vagally mediated. *Brain Research Bulletin*.

[30] Fleshner M, Silbert L, Deak T (1997). TNF-*α*-induced corticosterone elevation but not serum protein or corticosteroid binding globulin reduction is vagally mediated. *Brain Research Bulletin*.

[31] Fleshner M, Goehler LE, Schwartz BA (1998). Thermogenic and corticosterone responses to intravenous cytokines (IL-1*β* and TNF-*α*) are attenuated by subdiaphragmatic vagotomy. *Journal of Neuroimmunology*.

[32] Wieczorek M, Dunn AJ (2006). Effect of subdiaphragmatic vagotomy on the noradrenergic and HPA axis activation induced by intraperitoneal interleukin-1 administration in rats. *Brain Research*.

[33] Berthoud H-R, Neuhuber WL (2000). Functional and chemical anatomy of the afferent vagal system. *Autonomic Neuroscience*.

[34] Cunningham ET, Sawchenko PE (1988). Anatomical specificity of noradrenergic inputs to the paraventricular and supraoptic nuclei of the rat hypothalamus. *Journal of Comparative Neurology*.

[35] Vonck K, Boon P, van Roost D (2007). Anatomical and physiological basis and mechanism of action of neurostimulation for epilepsy. *Acta Neurochirurgica, Supplementum*.

[36] Henry TR, Bakay RAE, Pennell PB, Epstein CM, Votaw JR (2004). Brain blood-flow alterations induced by therapeutic vagus nerve stimulation in partial epilepsy: II. Prolonged effects at high and low levels of stimulation. *Epilepsia*.

[37] Hosoi T, Okuma Y, Nomura Y (2000). Electrical stimulation of afferent vagus nerve induces IL-1*β* expression in the brain and activates HPA axis. *American Journal of Physiology—Regulatory Integrative and Comparative Physiology*.

[38] Borovikova LV, Ivanova S, Nardi D (2000). Role of vagus nerve signaling in CNI-1493-mediated suppression of acute inflammation. *Autonomic Neuroscience*.

[39] Paxinos G, Watson C (1998). *The Rat Brain in Stereotaxic Coordinates*.

[40] Wrona D, Trojniar W (2005). Suppression of natural killer cell cytotoxicity following chronic electrical stimulation of the ventromedial hypothalamic nucleus in rats. *Journal of Neuroimmunology*.

[41] Keppler D, Decker K, Bergmeyer HU, Bergmeyer J, Grab M (1984). Metabolites. *Methods of Enzymatic Analysis*.

[42] Hernandez ME, Martinez-Fong D, Perez-Tapia M, Estrada-Garcia I, Estrada-Parra S, Pavón L (2010). Evaluation of the effect of selective serotonin-reuptake inhibitors on lymphocyte subsets in patients with a major depressive disorder. *European Neuropsychopharmacology*.

[43] Besedovsky H, del Rey A, Lajtha A (2008). Brain Cytokines as integrators of the immune-neuroendocrine network. *Handbook of Neurochemistry and Molecular Neurobiology*.

[44] Sapolsky RM, Plotsky PM (1990). Hypercortisolism and its possible neural bases. *Biological Psychiatry*.

[45] Novakova L, Haluzik M, Jech R, Urgosik D, Ruzicka F, Ruzicka E (2011). Hormonal regulators of food intake and weight gain in Parkinson's disease after subthalamic nucleus stimulation. *Neuroendocrinology Letters*.

[46] Seifried C, Boehncke S, Heinzmann J (2013). Diurnal variation of hypothalamic function and chronic subthalamic nucleus stimulation in parkinson's disease. *Neuroendocrinology*.

[47] de Koning PP, Figee M, Endert E, Storosum JG, Fliers E, Denys D (2013). Deep brain stimulation for obsessive-compulsive disorder is associated with cortisol changes. *Psychoneuroendocrinology*.

[48] Ballanger B, Jahanshahi M, Broussolle E, Thobois S (2009). PET functional imaging of deep brain stimulation in movement disorders and psychiatry. *Journal of Cerebral Blood Flow and Metabolism*.

[49] Kandel ER, Schwartz JH, Jessell TM (2000). *Principles of Neural Science*.

[50] Elenkov IJ (2004). Glucocorticoids and the Th1/Th2 balance. *Annals of the New York Academy of Sciences*.

[51] Viswanathan M, Pilo B, George JC, Etches RJ (1987). Effects of vagotomy on circulating levels of catecholamines and corticosterone in the pigeon. *Comparative Biochemistry and Physiology C*.

[52] Bugajski AJ, Zurowski D, Thor P, Ģdek-Michalska A (2007). Effect of subdiaphragmatic vagotomy and cholinergic agents in the hypothalamic-pituitary-adrenal axis activity. *Journal of Physiology and Pharmacology*.

[53] Thrivikraman KV, Zejnelovic F, Bonsall RW, Owens MJ (2013). Neuroendocrine homeostasis after vagus nerve stimulation in rats. *Psychoneuroendocrinology*.

[54] Carbia-Nagashima A, Arzt E (2004). Intracellular Proteins and Mechanisms Involved in the Control of gp130/JAK/STAT Cytokine Signaling. *IUBMB Life*.

[55] Schindler R, Mancilla J, Endres S, Ghorbani R, Clark SC, Dinarello CA (1990). Correlations and interactions in the production of interleukin-6 (IL-6), IL-1, and tumor necrosis factor (TNF) in human blood mononuclear cells: IL-6 suppresses IL-1 and TNF. *Blood*.

[56] Gil K, Bugajski A, Kurnik M, Thor P (2013). Electrical chronic vagus nerve stimulation activates the hypothalamic-pituitary-adrenal axis in rats fed high-fat diet. *Neuroendocrinology Letters*.

[57] Wasinski F, Bacurau RF, Moraes MR (2013). Exercise and caloric restriction alter the immune system of mice submitted to a high-fat diet. *Mediators of Inflammation*.

[58] Ebner K, Muigg P, Singewald N (2013). Inhibitory function of the dorsomedial hypothalamic nucleus on the hypothalamic-pituary-adrenal axis response to an emotional stressor but not immune challenge. *Journal of Neuroendocrinology*.

